# Complete Genome Sequence of the Probiotic Lactic Acid Bacterium *Lactobacillus Rhamnosus*

**DOI:** 10.5195/cajgh.2013.113

**Published:** 2014-01-24

**Authors:** Samat Kozhakhmetov, Almagul Kushugulova, Adil Supiyev, Indira Tynybayeva, Ulykbek Kairov, Saule Saduakhasova, Gulnara Shakhabayeva, Kenzhebulat Bapishev, Talgat Nurgozhin, Zhaxybay Zhumadilov

**Affiliations:** 1 Center for Life Sciences, Nazarbaev University, Astana, Kazakhstan; 2 Medical Social Institution for Older People and Invalids, Astana, Kazakhstan

**Keywords:** lactobacilli, lactic acid bacteria, genome sequencing

## Abstract

**Introduction:**

*Lactobacilli* are a bacteria commonly found in the gastrointestinal tract. Some species of this genus have probiotic properties. The most common of these is *Lactobacillus rhamnosus*, a microoganism, generally regarded as safe (GRAS). It is also a homofermentative L-(+)-lactic acid producer. The genus *Lactobacillus* is characterized by an extraordinary degree of the phenotypic and genotypic diversity. However, the studies of the genus were conducted mostly with the unequally distributed, non-random choice of species for sequencing; thus, there is only one representative genome from the *Lactobacillus rhamnosus* clade available to date. The aim of this study was to characterize the genome sequencing of selected strains of *Lactobacilli*.

**Methods:**

109 samples were isolated from national domestic dairy products in the laboratory of Center for life sciences. After screaning isolates for probiotic properties, a highly active *Lactobacillus spp* strain was chosen.

Genomic DNA was extracted according to the manufacturing protocol (Wizard® Genomic DNA Purification Kit). The *Lactobacillus rhamnosus* strain was identified as the highly active *Lactobacillus* strain accoridng to its morphological, cultural, physiological, and biochemical properties, and a genotypic analysis.

**Results:**

The genome of *Lactobacillus rhamnosus* was sequenced using the Roche 454 GS FLX (454 GS FLX) platforms. The initial draft assembly was prepared from 14 large contigs (20 all contigs) by the Newbler gsAssembler 2.3 (454 Life Sciences, Branford, CT).

**Conclusion:**

A full genome-sequencing of selected strains of lactic acid bacteria was made during the study.

